# Spatial Diffusion of Influenza Outbreak-Related Climate Factors in Chiang Mai Province, Thailand

**DOI:** 10.3390/ijerph9113824

**Published:** 2012-10-24

**Authors:** Supachai Nakapan, Nitin Kumar Tripathi, Taravudh Tipdecho, Marc Souris

**Affiliations:** 1 Remote Sensing and GIS Field of Study, School of Engineering and Technology, Asian Institute of Technology, P.O. Box 4, Klong Luang, Pathumthani 12120, Thailand; Email: nitinkt@ait.ac.th (N.K.T.); taravudh@ait.ac.th (T.T.); 2 Institut de Recherche pour le Développement (IRD), UMR 190, Marseille 13001, France; Email: souris@ird.fr

**Keywords:** influenza, spatial statistics, climate factors, climate variability, Geographic Information Systems (GIS), health risk map

## Abstract

Influenza is one of the most important leading causes of respiratory illness in the countries located in the tropical areas of South East Asia and Thailand. In this study the climate factors associated with influenza incidence in Chiang Mai Province, Northern Thailand, were investigated. Identification of factors responsible for influenza outbreaks and the mapping of potential risk areas in Chiang Mai are long overdue. This work examines the association between yearly climate patterns between 2001 and 2008 and influenza outbreaks in the Chiang Mai Province. The climatic factors included the amount of rainfall, percent of rainy days, relative humidity, maximum, minimum temperatures and temperature difference. The study develops a statistical analysis to quantitatively assess the relationship between climate and influenza outbreaks and then evaluate its suitability for predicting influenza outbreaks. A multiple linear regression technique was used to fit the statistical model. The Inverse Distance Weighted (IDW) interpolation and Geographic Information System (GIS) techniques were used in mapping the spatial diffusion of influenza risk zones. The results show that there is a significance correlation between influenza outbreaks and climate factors for the majority of the studied area. A statistical analysis was conducted to assess the validity of the model comparing model outputs and actual outbreaks.

## 1. Introduction

The geographical and seasonal distributions of many infectious diseases are linked to climate; therefore there exists the possibility of using seasonal climate forecasts as predictive indicators for disease outbreaks [1]. In addition, convincing evidence that anthropogenic influences are causing the World’s climate to change has provided an added incentive for improving our understanding of climate-disease interactions.

The existence of seasonal patterns and the climatic factor sensitivities of infectious diseases have been established; an important concern is the boundary at which changes in disease patterns will occur under global climate change conditions [2]. The effect of climate variability on infectious diseases is determined by the transmission cycles of each pathogen. The transmission cycle requirements of a vector or non-human host are typically more susceptible to external environmental inﬂuences than those diseases which only involve a pathogen and humans [3,4]. The factors of climate variability—minimum temperature, maximum temperature, humidity and rainfall—are major causes of seasonal diseases that impact socio-economics and public health [5]. Therefore, there is a need to understand the possible relationships between climate variability and cyclic epidemics in areas where there are climate connections. There may be great potential in applying the rapidly advancing science of seasonal (or El Niño) forecasting to the evaluation of disease risk and the health sector will be able to make good use of this information in health service planning [6].

Seasonal disease patterns can be influenced by increased variability and sustained changes in temperatures, rainfall patterns, flooding or droughts and rising of the sea level [7]. Influenza is one of the most significant diseases in humans, considered to be associated with approximately 250,000–500,000 global deaths every year [8]. Risk factors for influenza epidemics have been attributed to basic geographic receptivity governed by climatic conditions. Therefore, the effects of the ambient environment and a variety of environmental conditions have been considered an important factor related to the seasonality of influenza and its ability to cause epidemics in human hosts [8,9,10].

Influenza is one of the leading causes of mortality in developing countries [11,12]. It is a highly contagious acute respiratory disease that in the past has caused global epidemics and pandemics [13]. It can cause mild to severe illness, and can lead to death. While most healthy people recover from the influenza without complications, some people, such as the elderly, young children, and people with certain other health conditions, are at high risk for serious complications resulting from influenza. Human influenza is a disease that is transmitted from person-to-person via fluid droplets generated by coughing. The virus is stable in a dried condition and is thus also thought to be transmitted by droplet nuclei, which are produced after evaporation of water before falling to the floor [14].

Influenza pandemics also seem to be dependent on environmental or climate conditions [15,16], which may influence the onset, magnitude and duration of the influenza season. Climate could affect to the abundance of virus reservoirs, reactivate latent infections, the virulence of circulation strains relative to population immunity, or virus survival outside of the human body. In addition, climate may affect human-human contact patterns, susceptibility and infectiousness. The ability to predict epidemic patterns using climate forecasts could thus have important public health implications.

In Thailand, The Bureau of Epidemiology, Department of Disease Control of the The Ministry of Public Health has typically reported more than 20,000 influenza cases (per 100,000 populations) every year during the 2001–2008 period (42,371, 39,960, 29,918, 21,351, 21,176, 17,424, 18,368 and 20,881 cases, respectively). In 2007, the incidence of influenza corresponded with the highest number of deaths (15 people) [17].

**Figure 1:**
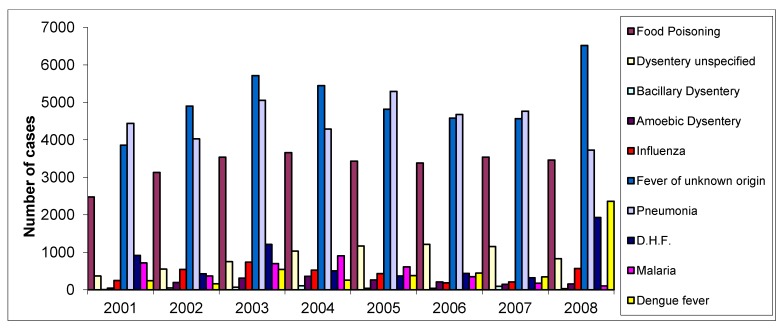
The top 10 diseases with the highest incidence during 2001–2008.

Chiang Mai Province has had influenza on its list of top 10 diseases with the highest incidence for many years (Figure 1). As indicated by the numbers of cases, the pattern of influenza epidemics in Chiang Mai varied from year-to-year between 2001–2008. The highest number was recorded in 2003 (731 cases), particularly in the months of June and July, with incidence rates of 96 and 112, respectively. The incidence of influenza displayed high variability during the late hot season and early rainy season. Climate factors (rainfall, temperature and humidity) [18], socio-demographic factors (age, occupation, *etc*.) are all thought to be related to the incidence and spatial distribution of influenza [19].

Epidemiological studies of influenza which examine the relationship between influenza incidence and the impacts of climate factors in Thailand are scarce for several reasons [20]. One of the reasons is that the data collected from patients with light symptoms were not considered by the health system. Influenza also can be confused with other diseases if the patients’ admissions to hospital were based on different symptoms, so the diagnostics and classifications for disease cases were often not correctly included in the epidemiology reports. Another reason may be the fact that it is difficult to determine the factors responsible for disease outbreaks and their relationship with the environmental factors, like climate. Of all the factors impacting influenza in Thailand, the climate factors are probably the least well understood. A better understanding of the relationship between climate factors and influenza incidence is essential for a study of potential climate change on influenza in the future.

Geographic Information Systems can be used to assess and identify potential risk factors involved in disease incidence and diffusion such as the variability factors. Spatial analysis and statistical tools such as temporal analysis are powerful tools for expressing a relationship and addressing the epidemic problem, allowing identification of risk areas and factors related to the modulation of disease dynamics [21]. Spatio-temporal relationships can provide for understanding of disease dynamics, in particular for influenza incidences, patterns and spreading dynamics [22]. Detection of spatial and temporal relations is useful for identifying risk areas for disease surveillance [23]. Exploration of the empirical relationships between major factors such as climate factors (temperature, rainfall, humidity) and the occurrence of influenza cases using multiple regression analysis has provided knowledge about the significant correlation between influenza and climate parameters in affected areas.

This study aimed to investigate the relationship between climatic factors and influenza cases and identify spatial patterns of influenza in Chiang Mai Province, Thailand. Spatio-temporal diffusion patterns of influenza cases also reveal previously unsuspected patterns leading to the formulation of additional theories based on the data of influenza cases from 2001 to 2008.

## 2. Materials and Methods

### 2.1. Physical and Climatic Setting of Chiang Mai Province, Thailand

#### 2.1.1. Physical Setting of Chiang Mai

**Figure 2:**
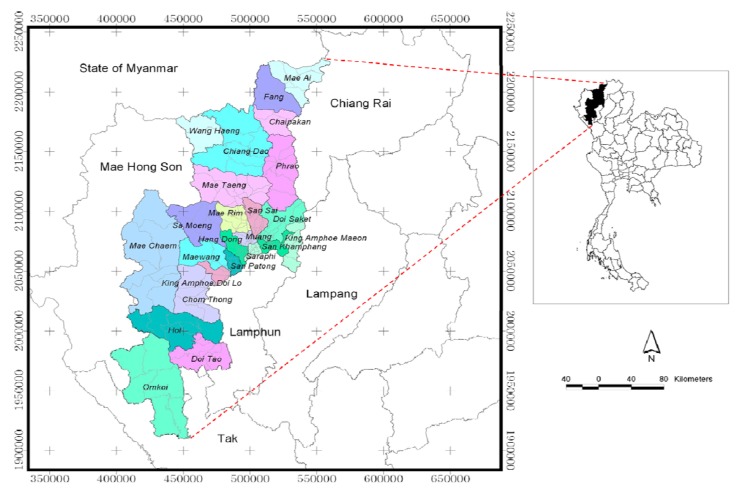
Study area: Chiang Mai Province, Thailand.

Chiang Mai, located between latitude 17°25'N to 20°15'N and longitude 98°00'E to 99°58'E (UTM: 390000E-560000E, 1900000N-2230000N) and covering an area of 22,061.17 km^2^, is the biggest province in northern Thailand (Figure 2). Its terrain mostly consists of forested mountains with an average elevation of 310 meters above mean sea level which generally run through the province in a north to south pattern. In 2006, the land utilization reports for the area indicated that the forested area is 76.97%, agricultural area 18.18%, urban and built up land are 2.96%, miscellaneous area is 1.05% and water body area is 0.84%.

Most of the area in the central part of the study area is a basin. It is a populated area used for business, residence and agriculture. Chiang Mai Province is divided administratively into 24 districts (amphoe), 204 sub-districts (tambon), and 2,070 villages (mooban). Chiang Mai has a population of about 1.6 million (figures for the 2001–2005 period are 1,600,850, 1,595,855, 1,603,220, 1,630,769 and 1,650,009, respectively). In 2006, the population was counted to be 1,658,298 persons, with the highest densities occurring in/around the Chiang Mai metropolitan area (*i*.*e*., Muang, Mae Rim, San Sai, Hang Dong, San Kamphaeng, Saraphi and San Pa Tong districts) [24].

Consequently, Chiang Mai Province was selected as a study area because of its high disease incidence. Climate obviously has a high spatial variability, so it is important that the observed climate factors be representative of the environmental conditions as experience by patients. In addition, Chiang Mai Province provides a high degree of variability of all climate factors, so this variability can provide a wide range of sample conditions for study. Thus, Chiang Mai Province has both a large enough population to provide consistent weekly disease incidences while the observed weather stations are sufficiently representative of environmental condition covered the study area.

#### 2.1.2. Climate Variability of Chiang Mai

Chiang Mai’s climate is subtropical, but is colder and less humid than other parts of Thailand. It can be clearly divided into three distinct seasons: hot, rain and cold seasons. The hot season runs from early March to the end of May, with a temperature range between 19 °C and 36 °C. During the daytime, the average temperature lurks somewhere around the 35 °C mark. The extreme high temperatures usually occur in April. The rainy season begins in early June and reaches to the end of October with the heaviest rainfall in August/September. The cold season lasts from late October to the end of February. During this period, the temperature ranges between 14 °C and 30 °C, and the nights are much colder. The coldest months are December/January.

### 2.2. Data Acquisition

#### 2.2.1. Epidemic Data

Daily reported sub-district (tambon) level morbidity case rate per 1,000 population data in Chiang Mai Province were collected. All the data were acquired from the Health Information System Section, Chiang Mai Provincial Public Health Office (CMPHO) [25]. The epidemiological data contained the daily numbers of patients with influenza cases at the tambon level in Chiang Mai from 2001 to 2008. The data represented only the patients for whom an official Form 506 was filled out at the visited hospital before reporting to the CMPHO [26]. The 506 forms contained each patient’s age, gender, address, and the dates of the hospital consultations [27].

#### 2.2.2. Climate Data

The climatic parameters for the years 2001–2008 covering Chiang Mai and Northern Thailand were obtained from the 13 meteorological stations of the Thailand Meteorological Department (TMD), Ministry of Information, Communication, and Technology, Thailand (MICT). The following climatic parameters from each station were included in this study to observe the change of climatic conditions: minimum/maximum temperature (°C), relative humidity (%) and rainfall (mm).

#### 2.2.3. Tambon Data

In this study, location data and population data for 204 sub-districts (tambons) of the province were collected from the Department of Provincial Administration, Thailand. Tambon point locations were confirmed for accuracy by overlaying on high resolution satellite images.

### 2.3. Spatio-Temporal Analysis

Influenza incidences separated by gender (male and female) and age groups were analyzed per year. The daily data of influenza cases and climate data (rainfall, temperature and relative humidity) for the years 2001–2008 were generated. Temporal patterns and correlation of influenza cases and climate data were also analyzed [28]. Influenza patterns in Chiang Mai Province appear to be closely related to the population increase. In the past, the disease was mainly restricted to the urban and semi-urban areas of the province, but over the years influenza has been spreading to rural areas, maybe due to population movement through transport development, economic activities and the changes in climate factors.

The numbers of influenza cases were determined as infected patient and geocode using village location from the treatment address in the hospital database. Mapping the spatial distribution of influenza cases by using an empirical Bayes smoothing (EBS) method which is based on the idea of pooling information across villages [29] overcomes the problem of a false view of reality due to a difference between the number of patients and the amount of population, when a small population density occurs generally in large areas. The influenza incidence rates per year or per month were adjusted by the EBS function and converted to the influenza morbidity rate (IMR).

The IDW interpolation technique is commonly used in GIS programs for producing surfaces using interpolation of scatter points such as rainfall point data and influenza transmission incidents. The technique is based on the assumption that the interpolating surface should be influenced mostly by the nearby points and less by the more distant ones [30]. The interpolated surface is a weighted average of the scatter points and has commonly been applied to climate data [31]. To produce a spatial distribution of influenza cases in Chiang Mai Province, Geographical Information Systems (GIS) techniques were used to register the maps of influenza incidence with topographical information at the divisional administrative unit level. Rainfall, temperature and relative humidity data interpolations were carried out using the IDW technique.

Moran’s Index is a simple translation of a non-spatial correlation measure to a spatial context and is usually applied to area unites where numerical ratio or interval data are available. Moran’s I can be defined simply as:


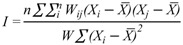


where *X_i_* is the value of the interval or ratio variable in area unit *i*. Other terms have been defined previously. The value of Moran’s I range from −1 for a negative spatial autocorrelation to 1 for a positive spatial correlation [32].

### 2.4. Multiple Regression Analysis

A wide range of adaptations of regression analysis have also been applied to the problem of modeling the distributions of diseases and their vectors. Multiple linear regressions analysis was established to explore and identify statistically significant risk indicators. The general purpose of multiple regressions is to learn more about the relationship between several independent or predictor variables and a dependent or criterion variable. It is then possible to construct a linear equation containing all the variables. Note that in this equation, the regression coefficients (or *b_0_*, *b_1_*, *b_2_*, … *b_k_*) represent the independent contributions of each independent variable to the prediction of the dependent variable. In a multiple regression model, a high variance explained (R-square) is expected. The higher the variance explained is, the better the model is. An important aspect of multiple regressions is the choice of the number of variables that go into the model. In general multiple regression procedures will estimate a linear equation of the form:

*Y* = *b_0_* + *b_1_X_1_* + *b_2_X_2_* + ∙∙∙ *b_k_X_k_*

where *k* is the number of predictors.

Multiple regression analysis allowed investigation of the predictive indicator of each of the factors [33]. The typical outcome of a multiple regression analysis is an equation or model that represents the set of independent variables for a particular dependent variable. The independent variable is used to predict changes in the dependent variable [34]. Long-term trends in both climate factors and influenza cases in the years 2001–2008 were adjusted and used as indicator variable to model in place of parameter terms. This model can be verified by using the *R^2^* statistic.

One way ANOVA and independent sampled *t-*tests were used to test the mean differences of influenza cases and monthly climate factors among three parameters: temperature difference, relative humidity and number of rainy days. Pearson’s correlation coefficient test was used to detect primary associations between influenza cases and climate factors. The result expresses the *p* value (<0.01 and <0.05) and was employed to identify statistically significant risk indicators.

## 3. Results

### 3.1. Spatio-Temporal Analysis of Influenza

#### 3.1.1. General Analysis

**Figure 3:**
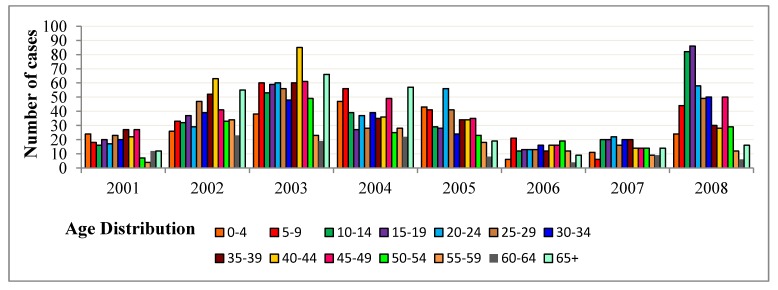
Number of influenza cases classified by age groups during the years 2001–2008.

The epidemiology data collected from 2001–2008 was classified into several demographic groups by gender, age groups and time, as shown in Figure 3. The highest recorded influenza incidence occurred in 2003, with 731 cases for that time period. After 2004, a gradual decrease was seen into the influenza cases until 2007, when another increase occurred. The lowest number of cases (182) occurred in 2006. A total of 3,447 cases were reported, including 1,739 male (50.01%) and 1,708 (49.99%) female patients.

The age distribution of influenza cases during 2001–2008 was high for the children (0–14 years) and adult (15–59 years) groups and low in the elderly (≥60 years) group (Figure 3). The highest number of influenza cases occurred in the 15–19 years of age group, with about 86 cases for the year 2008. There were no gender differences in the amount of influenza cases each year.

#### 3.1.2. Temporal Analysis

**Figure 4:**
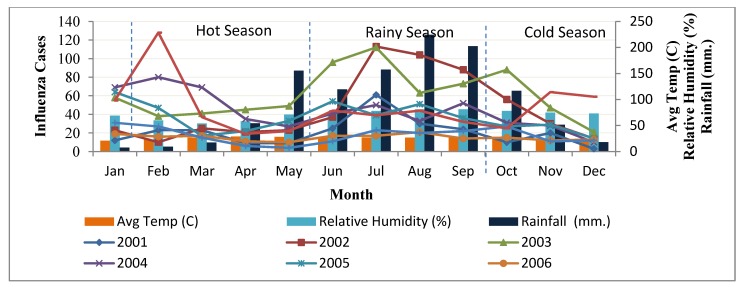
Number of influenza cases with average rainfall, relative humidity and temperature on monthly during the years 2001–2008.

The climate in Chiang Mai and Northern Thailand is characterized by the monsoon, which creates three distinct seasons. The south-west monsoon usually arrives from India at the end of May and lasts until November. Rainfall is generally heaviest in September, with an average precipitation of 250 mm for that month alone. The north-east monsoon lasts from mid-November until early May and brings cold air from northern Vietnam/China but no rain to Northern and Central Thailand. From March to May heat is on the agenda and the maximum daytime temperature ranges between 19 °C and 36 °C. During the daytime, the average temperature lurks somewhere around 40 °C/100 °F. Still, at night it mostly cools down and mornings can be quite pleasant, and the evenings balmy. The influenza incidence trend follows these three seasons every year (Figure 4).

**Figure 5:**
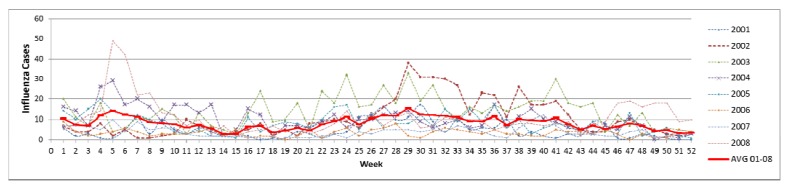
Weekly number of influenza cases during the years 2001–2008.

The influenza temporal distribution was normally high during the rainy season, presenting a similar trend each year. The disease patterns increase rapidly beginning in May and are highest in July before going down until October during the rainy season (Figure 5). The critical month with worst incidence was recorded in July with more than 100 cases. Influenza outbreaks generally occurred rapidly in the first part of rainy season because of the changing climate factors. Rainfall, temperature difference and relative humidity all start to change in May, consequently the highest influenza incidence was reported from the month of June to October which shows an increasing number of disease cases when rainfall and relative humidity are at their highest but temperature difference showed little change.

### 3.2. Multiple Regression Model Development

The variables used in the model are as follows:

*Dependent variable* is Case of incidence. Number of weekly influenza cases.

*Independent variables* are Temperature, Rainfall, and Relative Humidity.

Multiple regression analysis is employed to develop an empirical model to predict the influenza incidences [35]. The independent variables were used to predict changes in the dependent variable in the rainy and annual seasons. The variables used in the models are explained in the Table 1.

**Table 1:** Variables in the multiple regression models (weekly).

**Dependent Variable**			
*Y*		Estimated number of influenza cases	
**Independent Variable**			
*X_r_*		Amount of rainy day	
*X_t_*		Temperature difference	
*X_Rh_*		Relative humidity	

The independent variables were used to predict changes in the dependent variable in the rainy and non-rainy seasons. This model was verified using the *R^2^* statistics.

Influenza incidences per 1,000 populations in Chiang Mai Province varied from 1–49 cases with a mean and standard deviation of 8.27 ± 3.02 cases by week. From eight years’ data (*i*.*e*., 2001–2008), influenza incidence per 1,000 populations was used to determine the optimal climate conditions for influenza epidemics in Chiang Mai, and statistical analysis was derived using the most strongly correlated factors. The number of rainy days, temperature difference and relative humidity were examined for associations between climate factors and influenza incidence (Table 2).

**Table 2:** Mean ± SD of climate factors on Chiang Mai Province.

Climate Factors	Variable	Year	Rainy Season	*t-*test
Influenza cases	*Y*	8.27 ± 3.02	9.97 ± 2.43	*t*_1.52_ = −0.438 *
Rainy days	*X_r_*	2.21 ± 1.74	4.05 ± 0.66	*t*_1.52_ = 1.394 **
Temperature Difference	*X_t_*	11.05 ± 3.00	8.12 ± 0.81	*t*_1.52_ = 0.785 *
Relative Humidity	*X_Rh_*	74.12 ± 10.26	82.44 ± 2.35	*t*_1.52_ = 1.043 *

(* p < 0.05, ** p < 0.01)

Multiple linear regression models from two time periods as whole year and rainy season showed that there were three main climate factors associated with influenza cases: rainy days, temperature difference and relative humidity (Table 3).

**Table 3:** Linear regression models on climate factors and influenza cases in Chiang Mai Province.

Period	Multiple Regression Model	Linear Regression Model	*F* Value
Year	Relationship Equation-1(RE-1)	*Y* = 0.789*X_r_* + 0.420*X_t_* + 0.107*X_Rh_ −* 6.039	*F*_3.48_ = 2.51 *
Rainy Season	Relationship Equation-2(RE-2)	*Y* = −0.783*X_r_* – 2.663*X_t_* – 0.079*X_Rh_* + 41.299	*F*_3.17_ = 6.97 **

(* p < 0.05, ** p < 0.01)

#### 3.2.1. Relationship Equation-1 (RE-1)

The Relationship Equation-1 (RE-1) is a relationship between number of influenza cases and the climate factors (*X_r_*, *X_t_* and *X_Rh_*) during 8 years.

Therefore, the selected regression model was:

*Y* = 0.789*X_r_* + 0.420*X_t_* + 0.107*X_Rh_*− 6.039

**Figure 6:**
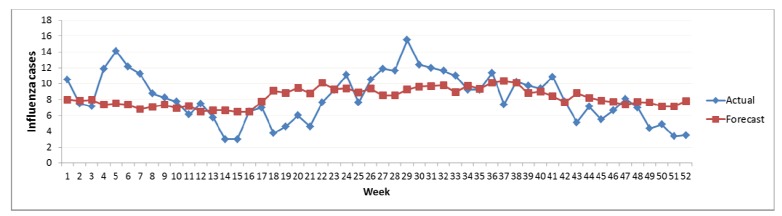
Relationship between actual and forecast influenza incidences RE-1.

The coefficient of determination (*R*^2^) was found to be 0.37 and validated with climate data as shown in Figure 6.

#### 3.2.2. Relationship Equation-2 (RE-2)

Multiple regression analysis was determined for the occurrences of influenza incidence during the rainy season. The Relationship Equation-2 (RE-2) between influenza incidences and climate factors was as follows:

Therefore, the selected regression model was:

*Y* = −0.783*X_r_* – 2.663*X_t_* – 0.079*X_Rh_* + 41.299

**Figure 7:**
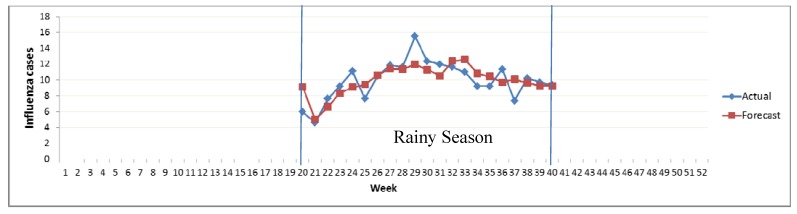
Relationship between actual and forecast influenza incidences RE-2.

The coefficient of determination (*R*^2^) was found to be 0.65 and was validated with climate data as shown in Figure 7. It was observed that the *R*^2^ for this model was low for the non-rainy season but high for the rainy season (Figure 6–Figure 7).

The results support the previous finding that there is a clear cyclical variability of influenza transmission in Chiang Mai. Most previous studies were done in temperate zones and showed that influenza exhibited distinct seasonality with a higher incidence in rainy months and sharp decrease of morbidity rates in the summer months [36]. This may be because during rainy weather, the differences between maximum and minimum temperatures increased and the number of influenza incidences increased [37]. This could be due to two possible reasons. When there are huge differences between the maximum and minimum temperatures (*i*.*e*., temperature differences), this may decrease host resistance [38]. Furthermore, climate factors such as humidity may play a role in the cyclical nature of influenza, along with the behavioral changes that coincide with more severe rains [2,39].

#### 3.2.3. Spatial Analysis

**Figure 8:**
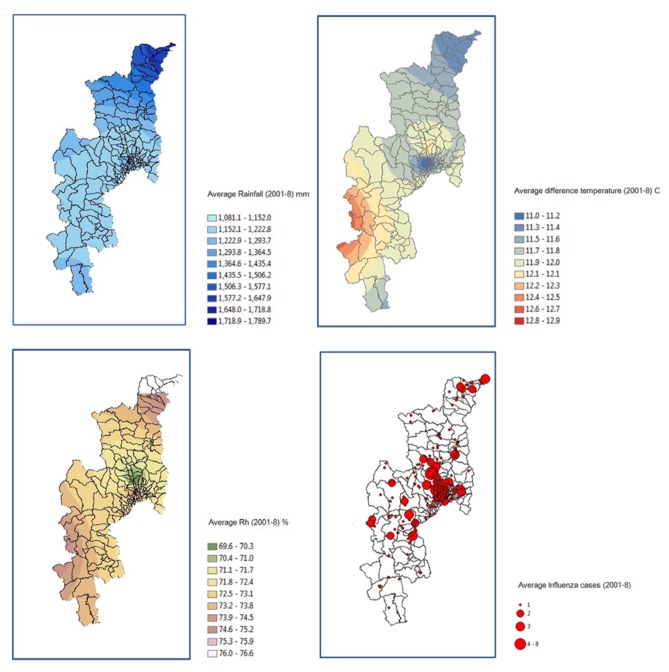
Spatial distribution of climate factors and influenza incidence: Chiang Mai Province.

Climate data from the meteorological stations produced using the IDW interpolation technique and influenza incidence occurrence showed that they are strong positive relationships between climate data and influence incidence (Figure 8). A well-defined seasonality in the epidemics was found, that allows us to conclude that colder temperatures and less abundant rainfall appeared to be influencing the epidemics. This study also showed that high influenza incidence is normally associated with the areas with less relative humidity. The global spatial autocorrelation analysis with Moran Index showed that the spatial distribution of Influenza Morbidity Rate (IMR) was clustered [40], for all the years 2001–2008 (Table 4). This informed a major finding that suggests to public health departments that influenza is occurring in clusters and not spread uniformly or randomly throughout the province. These locations may be considered as hotspots for future control strategies [41]. The highest of Moran’s I and G-statistic (Z-score) values were confirmed as 0.08 and 19.36, respectively, in the year 2008. It presented an expected clustered pattern for an infectious disease at the tambon level.

**Table 4:** Global spatial autocorrelation analysis of IMR.

Year	IMR		Pattern
	Moran’s I	Z (I)	
2001	0.02	6.26	Clustered
2002	0.08	19.08	Clustered
2003	0.07	16.08	Clustered
2004	0.06	14.76	Clustered
2005	0.08	18.12	Clustered
2006	0.08	18.11	Clustered
2007	0.05	12.18	Clustered
2008	0.08	19.36	Clustered

Note: the corresponding *p* < 0.01.

### 3.3. Influenza Risk Zone Map Detection

**Figure 9:**
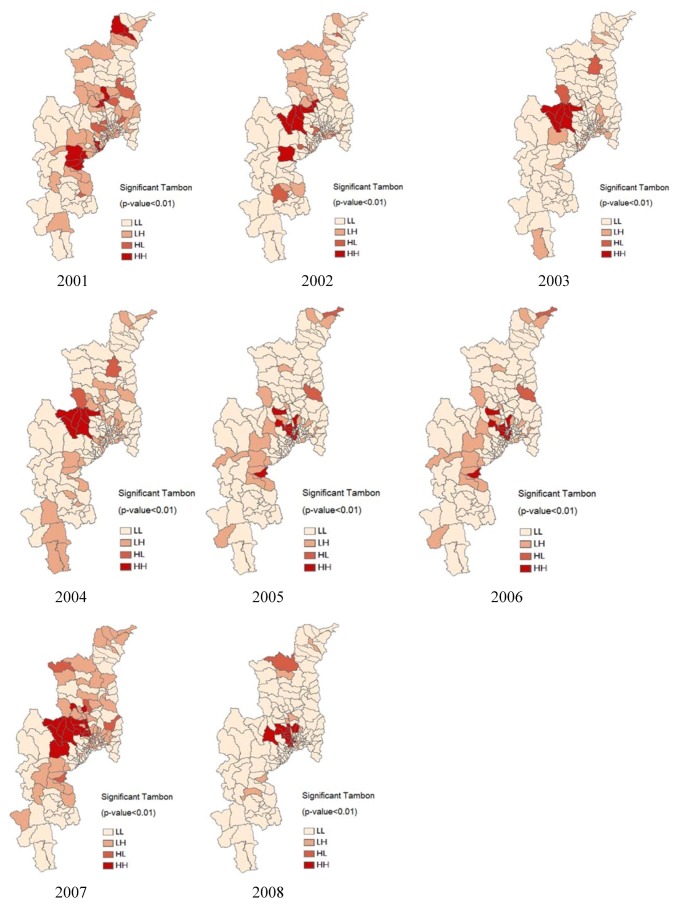
Risk map of influenza potential risk from 2001–2008.

This describes the methods for generating a potential risk map of influenza cases by considering both the location and their attributes [42]. Spatial clusters of influenza morbidity rate cover Chiang Mai Province. The results from 2001–2008 are presented. The Sub-Districts (tambon) level map with significant local indices of spatial association (*p *< 0.01) using the local Moran’s I statistic represents the influenza risk map. The standardized values of IMR in each tambon were displayed in spatial cluster plots, to contrast the observed value with their spatial average (spatially averaged adjacent values), and to detect outliers. The clustered tambons with high IMR (risk map) were thus found. The map in Figure 9 shows the locations with significant local Moran statistics and classifies those locations by type of association (cluster map).

The outputs represent the spatial autocorrelation of disease incidence at the tambon level. The study only focused on the unvariate spatial distribution and the location of any significant clusters or spatial outliers in the MBR data [43]. The locations of significant local Moran’s I statistics was classified by type of spatial association. The dark red (HH) and lightly pink (LL) locations were indications of spatial clusters (respectively, high surrounded by high, and low surrounded by low). In the other hand, the light red and lighter red were indications of spatial outliers (respectively, high surrounded by low (HL), and low surrounded by high (LH)) [44].

The potential risk maps of influenza disease during 2001–2008 were concentrated in the middle (Samoeng and Muang) districts of Chaing Mai during 2002–2004. A clustering of risk zones occurred within the area of Samoeng district for the years 2002–2004 and 2007 (I = 1.45–18.71), and Muang district in 2005–2006 (I = 0.82–18.12). The risk zone map of influenza during 2001–2008 was found and illustrated by overlaying the cluster maps, as shown in Figure 9. The maps of local spatial correlation indices were used to display the risk zones with red colored areas (high surrounded by high, respectively). These maps show clear spatial patterns of influenza that were concentrated in the middle (San Kamphaeng district) and north (Fang, Chai Prakan and Wiang Haeng districts) of Chiang Mai. The densest clustering of risk zones occurred within the urban areas of Samoeng district in 2002–2004.

## 4. Discussion

This study found that temporal distribution of influenza cases was closely associated with the climate factors during the rainy season period. The relationship was statistically supported by the multiple regression model established in this study indicating that there is a strong statistical association between influenza and rainy days, temperature difference and relative humidity. Influenza incidences were relatively high during the rainy days and decreased when the rainfall started to decrease. The temperature difference did not change much in a weekly period during the rainy seasons, while the influenza cases were increasing. Influenza incidence increases significantly when the relative humidity is also high. The outbreaks predicted by the model were clearly related to the actual outbreaks indicating its ability to predict potential outbreaks. The applicability of the model can be further tested with other respiratory diseases such as fever of unknown origin and pneumonia at different geographical regions in other countries.

The production of the influenza risk map in this study used the spatial distribution of influenza incidence, rainy day, temperature difference and relative humidity with the high risk population category. The spatial distribution of influenza risk shows that influenza incidence is clustered in the middle and northern part of Chiang Mai Province, mainly around the urbanized regions of the province (Figure 9).

To predict the cause of influenza incidence, a better understanding of associations between climate factors in the spread of influenza is essential. Various public health measures are now in place to prevent further outbreaks. However, in the event that subsequent outbreaks should occur, additional analysis of the underlying climatic conditions is needed to verify the seasonal nature of the disease. The advantage of using GIS-based methodology is its ability to incorporate diverse data and integrate expert knowledge using statistical techniques such as multi-criteria analysis. Using the GIS-based methodology, the future work can be undertaken to examine the impact of urbanization, climate variability on the changes of disease habitats incorporating remote sensing and climatic phenomena such as El Niño/La Niña data [45].

A strength of this study was that it explored statistical and spatial analysis to explain relationships between climate factors and influenza cases in the studied area [46,47]. More studies in this regard could perhaps reveal the strong correlation between the climatic changes and disease outbreaks, which would help in strategic planning to forecast more accurately any outbreaks and to deal with any future outbreaks well in advance. However, there were some limitations in the study due to administrative recognition. As different data sources, epidemiological data were unrecognized in some tambon levels. Therefore, the influenza cases data did not figure in earlier years. The incidence rate was set to 0; this might not available in the epidemiological reports and were not included as a determinant in this study.

## 5. Conclusions

This study identified and mapped the spatial distribution of influenza incidence, potential influenza risk based on influenza incidence, high risk population age groups, and developed a model to predict influenza outbreaks analyzing the association between climate factors (rainy days, temperature difference and relative humidity) and influenza outbreaks. The methodology developed using GIS, spatial statistics and multiple regression models has improved our understanding of disease outbreak patterns and its association with climatic changes. We found a temporal and spatial correlation between rainfall seasons and influenza disease outbreaks for the middle and north part of Chiang Mai Province based on an analysis of climate, population and epidemiology data obtained for the 2001–2008 period. A multiple regression model was constructed to assess the quantitative statistical relationships between climate factors and influenza incidence. At major urban centers (e.g., Samoeng and Muang districts), the multiple regression model could closely predict the disease outbreaks using the dependent climatic variables. Understanding the spatial and temporal patterns of climate and its impact on human health, particularly outbreaks of respiratory diseases such as influenza, is of great importance in controlling the transmission and outbreak of the disease and treating infected populations effectively. It is felt that the availability and analysis of daily climatic and disease data may provide further insight and greater understanding in the future for better healthcare and disease control.
